# Correlation between CdSe QD Synthesis, Post-Synthetic Treatment, and BHJ Hybrid Solar Cell Performance

**DOI:** 10.3390/nano6060115

**Published:** 2016-06-14

**Authors:** Michael Eck, Michael Krueger

**Affiliations:** 1 Department of Microsystems Engineering (IMTEK), University of Freiburg, Georges-Köhler-Allee 103, 79110 Freiburg, Germany; eck@imtek.de; 2 Department of Physics, Carl von Ossietzky University of Oldenburg, Carl-von-Ossietzky-Straße 9-11, 26129 Oldenburg, Germany

**Keywords:** bulk heterojunction solar cells, hybrid solar cells, nanocrystals, quantum dots, conjugated polymers, post-synthetic treatment, thermal annealing, long-term stability

## Abstract

In this publication we show that the procedure to synthesize nanocrystals and the post-synthetic nanocrystal ligand sphere treatment have a great influence not only on the immediate performance of hybrid bulk heterojunction solar cells, but also on their thermal, long-term, and air stability. We herein demonstrate this for the particular case of spherical CdSe nanocrystals, post-synthetically treated with a hexanoic acid based treatment. We observe an influence from the duration of this post-synthetic treatment on the nanocrystal ligand sphere size, and also on the solar cell performance. By tuning the post-synthetic treatment to a certain degree, optimal device performance can be achieved. Moreover, we show how to effectively adapt the post-synthetic nanocrystal treatment protocol to different nanocrystal synthesis batches, hence increasing the reproducibility of hybrid nanocrystal:polymer bulk-heterojunction solar cells, which usually suffers due to the fluctuations in nanocrystal quality of different synthesis batches and synthesis procedures.

## 1. Introduction

**Figure 1:**
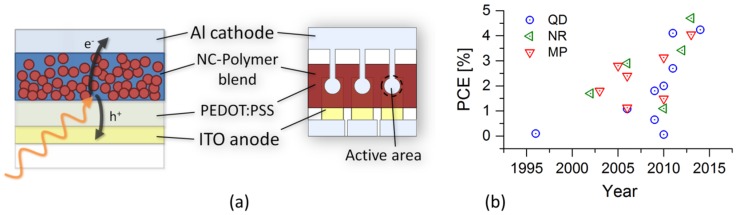
(**a**) Working principle and device structure shown on the cross-sectional and on the top view of the investigated hybrid bulk heterojunction (BHJ) solar cell with the active area size of 0.07 cm^2^. (**b**) Power conversion efficiencies of selected hybrid BHJ solar cells published over the years from nanocrystals (NCs) of different shape: quantum dots (QD) [2,3,6,7,8,9,10,11,12], nanorods (NR) [5,10,13,14,15], multipods (MP) [4,10,16,17,18,19,20].

Solar cells possessing a photoactive layer made out of semiconducting nanocrystals (NCs) and a conjugated polymer blend are called hybrid bulk heterojunction (BHJ) solar cells. Their photoactive layer typically possesses a thickness of around 100 nm, and is deposited from a NC/polymer dispersion [1]. A typical device structure is shown in Figure 1a. Within the photoactive layer, the polymer is mostly utilized as donor material for the electrons arising from the photo-induced excitons, while the NCs serve as electron acceptors and extraction material. Already the seminal work of Greenham *et al.* [2] published in 1996 has acknowledged the crucial negative impact of the insulating NC synthesis ligands on the solar cell performance, and a post-synthetic pyridine treatment of NCs was reported to improve overall solar cell performance. Since then, hybrid BHJ solar cells have undergone a remarkable development over the years (see Figure 1b), now exceeding efficiencies of 4% [3,4,5,6]; the currently best hybrid solar cells using NCs of different shapes have in common the usage of the low-bandgap polymer PCPDTBT (poly[2,6-(4,4-bis-(2-ethylhexyl)-4H-cyclopenta[2,1-b;3,4-b’]dithiophene)-alt-4,7-(2,1,3-benzothiadiazole)]) and of CdSe as NC material. Moreover, the best solar cells of the respective NC shape (spherical [6], elongated [5], multibranched [4]) all utilize the attachment of a thiol group to the CdSe NC surface. Thereby, high open circuit voltages of 0.71–0.74 V are achieved, leading to power conversion efficiencies (PCE) of 4.05%–4.7%.

Nevertheless, despite almost reaching the PCEs of (poly[2,6-(4,4-bis-(2-ethylhexyl)-4H-cyclopenta[2,1-b;3,4-b’]dithiophene)-alt-4,7-(2,1,3-benzothiadiazole)]) (PCPDTBT):fullerene-based BHJ solar cells, which are up to 5.24%–5.5% [21,22], the utilization of NCs as electron acceptors has the aforementioned disadvantage of requiring post-synthetic NC treatment to reduce and or exchange the initial synthesis ligands. This treatment, however, may result in the partial destruction of the NC surface [23]—leading to increased charge recombination—and to the formation of NC aggregates, which might introduce shortcuts over the thin active layer. Hence, several publications on hybrid BHJ solar cells report the filtration of the post-synthetically treated NC dispersion, in order to remove large NC aggregates [4,20]. The approach we follow is to avoid NC aggregation within the process of post-synthetic NC treatment prior to incorporation into the polymer:NC blend dispersion. We achieve this by a gradual reduction of the NC ligand sphere [11,15,24] through a protonation step of the hexadecylamine (HDA) capping ligands with hexanoic acid (HA) and their subsequent solvation with the polar solvent methanol. We observed an influence from the post-synthetic treatment duration on the NC ligand sphere size (see Figure 4). Therefore, we believe that we are able to minimize the post-synthetically induced number of NC surface defects, which is supported by the excellent ideality factor of 1.22–1.33 for our CdSe/PCPDTBT solar cells [6], which is relatively close to unity compared to other hybrid BHJ solar cells [25,26,27,28], even when compared to organic BHJ photovoltaic cells [29,30].

## 2. Results and Discussion

### 2.1. NC Synthesis

**Figure 2:**
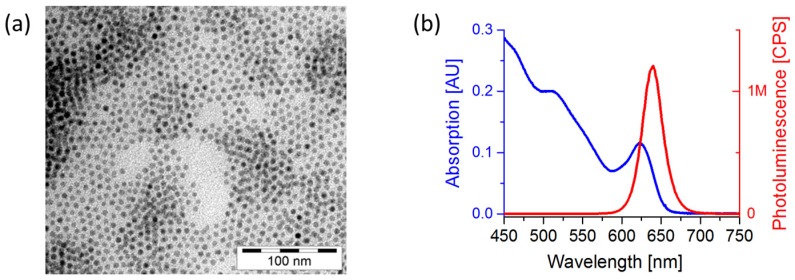
(**a**) Transmission electron microscopy (TEM) image of CdSe nanocrystals (NCs) used for the investigation from a 100:3:2 (hexadecylamine/trioctylphosphine oxide):(cadmium-stearic acid):(trioctylphosphine-selenid) ((HDA/TOPO):(Cd-SA):(TOP-Se)) ratio, synthesized for 30 min at 300 °C by wet-chemical hot injection NC synthesis with an average diameter of 6.5 nm. (**b**) Ultraviolet-visible (UV-Vis) absorption spectrum (blue line) and photoluminescence spectrum of CdSe nanocrystals (NCs) synthesized by a hot injection method recorded at an excitation of 575 nm (red line).

CdSe QDs, capped with HDA and trioctylphosphine oxide (TOPO) ligands, were synthesized according to Yuan *et al.* [31]. An exemplary transmission electron microscopy (TEM) picture of the spherical CdSe NCs with a typical diameter of about 6.5 nm as well as the ultraviolet-visible (UV-Vis) absorption and photoluminescence (PL) spectrum are given in Figure 2.

**Figure 3:**
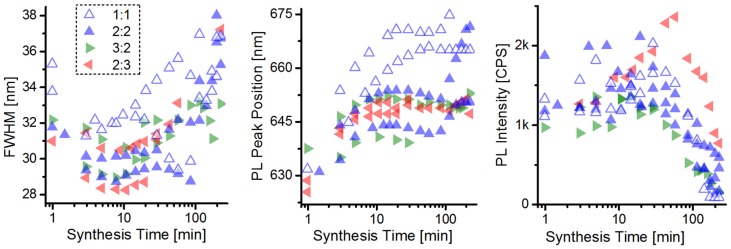
Different Cd:Se precursor ratios and concentrations—full width at half maximum (FWHM), photoluminescence (PL) peak position, and PL intensity evolution during the NC syntheses.

In order to increase the NC size uniformity, the precursor concentration was increased from a 100:1:1 (hexadecylamine/trioctylphosphine oxide):(cadmium-stearic acid):(trioctylphosphine-selenid) (HDA/TOPO:Cd-SA:TOP-Se) molar ratio to a 100:2:2 ratio [24]. Furthermore, to study the influence of unintentionally occurring fluctuations of practically utilized precursor ratios for the NC synthesis, the Cd:Se precursor ratio was varied from 3:2, over 2:2, to 2:3. The parameters of PL intensity, PL peak position, and full width at half maximum (FWHM) were measured and compared to aliquots taken during the NC synthesis at different reaction times and are depicted in Figure 3.

From the parameters of the CdSe quantum dots (QDs)—determined from extracted samples during 2 different syntheses for each precursor ratio—shown in Figure 3, one can observe that indeed a lower minimal FWHM is observed when using higher precursor concentrations. Moreover, a tendency of lower minimum FWHM is also observed for the increased Se precursor concentration; this synthesis furthermore exhibits a higher PL quantum yield in the synthesis brightpoint (point where the PL intensity reaches its maximum during synthesis). These observations confirm the findings firstly described by Qu *et al.* [32]. The properties of CdSe QDs in their brightpoint of different precursor concentrations—utilized for BHJ hybrid solar cell fabrication—are summarized in Table 1.

### 2.2. Post-Synthetic NC Treatment

**Figure 4:**
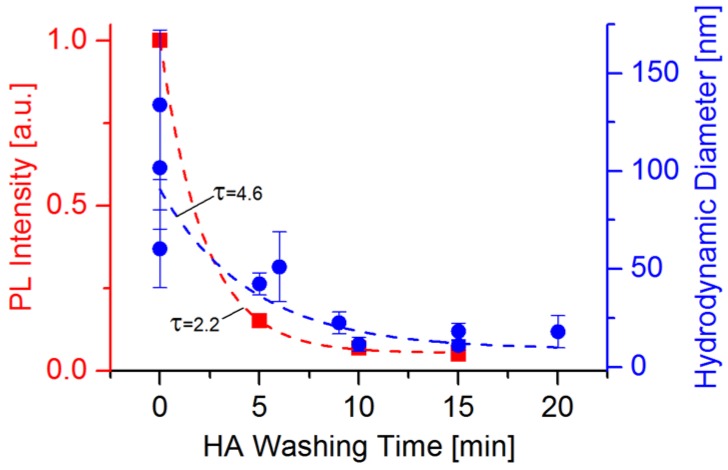
Red squares: Development of PL intensity over the HA washing time for CdSe QDs synthesized in hexadecylamine/trioctylphosphine oxide (HDA/TOPO) (100:3:2, 30 min at 300 °C). Blue dots: Respective development of the hydrodynamic diameter together with the standard deviation for CdSe QDs measured by dynamic light scattering (DLS). For both, the half-life τ in min is given for the respective fitted exponential decay.

As mentioned before, CdSe QDs were taken from the PL brightpoint, since we expect an optimal NC quality in this point, according to Qu *et al.* [32]. A hexanoic acid-based treatment was applied on CdSe QDs synthesized at three different Cd:Se ratios as described in the methods section. As we have presented in previous publications [11,15,33], the NC possesses a sphere of weakly associated ligands, which crystallize after the cool-down of the synthesis reaction around the initial ligand layer chemisorbed to the NC surface. To demonstrate the physical influence of the post-synthetic treatment on the CdSe QDs, the PL intensity and the ligand sphere size were determined for different hexanoic acid (HA) washing times (Figure 4). The peak PL intensity was determined from PL spectroscopy measurements of re-dispersed CdSe QDs, while the ligand sphere size was determined by measuring the hydrodynamic QD diameter (corresponding to the ligand sphere size [34]) by dynamic light scattering (DLS) of the same samples.

It can be seen that both the hydrodynamic diameter and the PL intensity seem to decrease exponentially with increasing HA washing time, corresponding to the facile removal of the outer, weakly associated ligand shell. The DLS measurements of untreated QD samples show a relatively wide value distribution, which might also partially result from QD aggregation to superstructures. Nevertheless, from the DLS measurements we can draw the conclusion that the NC ligand sphere diameter is decreasing with increasing HA washing time, while from the PL intensity measurements one can assume that the original ligand passivation of the NC surface is either reduced or changed (*i.e.*, by a ligand exchange with HA and/or MeOH).

### 2.3. Optimal Initial Solar Cell Performance

**Figure 5:**
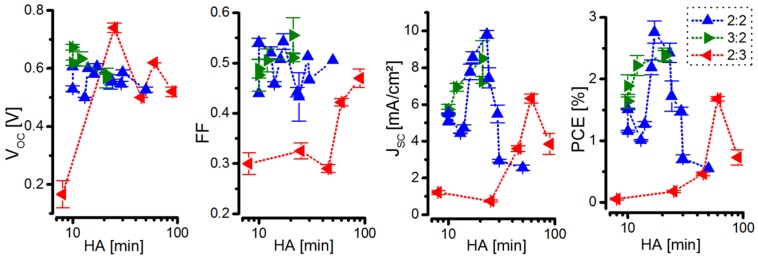
Dependency of open-circuit voltage (*V*_OC_), fill factor (FF), short-circuit current density (*J*_SC_) and PCE on the HA washing time at 105 °C for hybrid BHJ solar cells containing CdSe NCs taken from the respective PL brightpoint of the NC hot-injection syntheses performed at 300 °C with a 100:3:2, 100:2:2, and 100:2:3 ratio of (HDA/TOPO):(Cd-SA):(TOP-Se). The results are obtained from two synthesis batches for each ratio and from 60 solar cells, which were thermally post-annealed until reaching their optimal performance.

Using CdSe QDs taken from the brightpoint of the respective NC synthesis (*i.e.*, from 21 min synthesis time for the 100:2:2 ratio, 16 min for 100:3:2, and 60 min for 100:2:3), hybrid BHJ Solar cells were fabricated and characterized according to the methods section. In order to obtain the best solar cell performance, the optimal post-synthetic NC treatment time had to be found (see Figure 5).

From these experiments the post-synthetic NC treatment times, resulting in the optimal hybrid solar cell power conversion efficiency (PCE), were determined for 3 differently synthesized QDs. A summary of the optimal NC treatment time based on the experiments presented in Figure 5, the resulting average optimal PCE and the initial properties of the utilized QDs are presented in Table 1.

The parameters summarized in Table 1 show that there is a correlation in between the initial PL QY of CdSe QDs, and the required post-synthetic treatment time for creating the most efficient hybrid solar cell: a high PL QY requires a long post-synthetic NC treatment time. Furthermore, one can see that low PL FWHM leads to high PCEs for BHJ hybrid solar cells. Hence, the PL QY is an indicator of the required post-synthetic treatment time but not for the achievable solar cell performance. This is determined—given that the post-synthetic treatment is optimized—by the FWHM for comparable NC sizes, as they were in this investigation. Lower FWHM results in a higher PCE, and higher FWHM results in a lower PCE. This is reasonable, since a low FWHM presumably corresponds to a high intrinsic uniform NC quality.

### 2.4. Influence of the Post-Synthetic NC Treatment on the Device Annealing Time, and Guidance for NC Treatment

**Figure 6:**
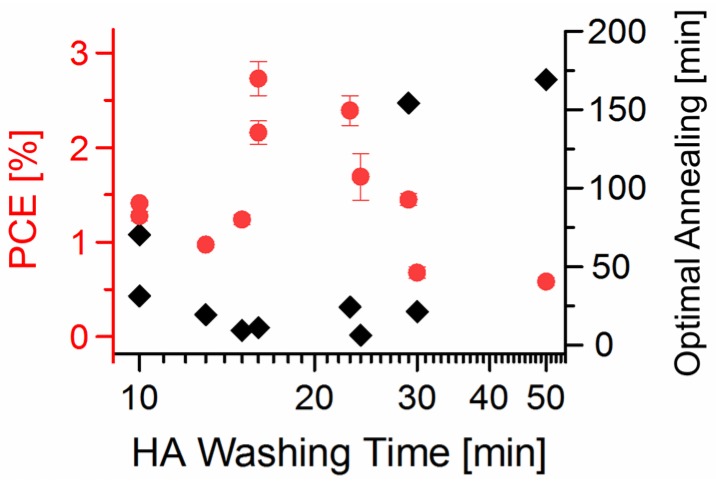
Power conversion efficiencies (PCEs) (red spherical points) obtained from 36 hybrid BHJ solar cells containing CdSe QDs (100:2:2) washed for different times in HA, and the optimal annealing time (black rhombic points) required to reach this efficiency.

To obtain the optimal solar cell performances presented in the prior paragraph, besides the optimal NC treatment time, an optimal time for the thermal solar cell annealing is also necessary. This optimal annealing time is however different for different NC post-synthetic treatment times as shown in Figure 6.

**Figure 7:**
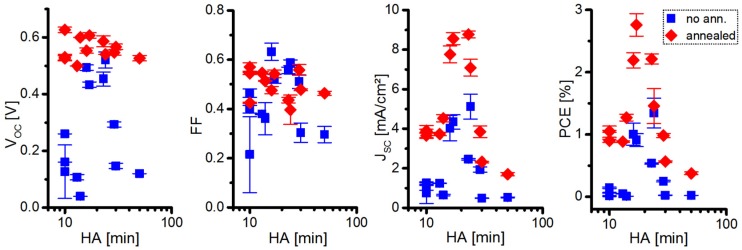
Differently long HA washed CdSe QDs (100:2:2) exhibit different performances when incorporated inside hybrid BHJ solar cells both without annealing and with 10 min of thermal annealing at 145 °C (results obtained from 36 cells).

**Figure 8:**
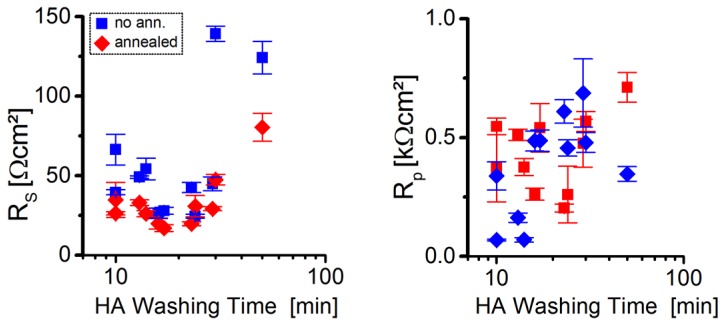
Incorporating differently long HA washed CdSe QDs (100:2:2) into BHJ hybrid solar cells results in different series resistance (*R_S_*) and parallel resistance (*R_P_*) values before and after 10 min of thermal annealing.

When taking a closer look at this behavior (see Figure 7 and Figure 8), one can even identify indications for finding the optimal NC treatment time with a reduced number of initial experiments.

As depicted in Figure 7, the short-circuit current density and the open-circuit voltage are already relatively high, near the optimal HA treatment time, even before the thermal annealing step. This is since the series resistance—representing the active layer resistivity and to a lesser extent also the contact resistances [35]—has its lowest values for longer HA washing times (see Figure 8). The reason for this is probably the decreasing NC ligand sphere diameter, measured during the first 15 min of the NC treatment (see Figure 4), leading to an increased conductivity of the QD phase within the active layer and a more intimate NC packing, improving electron hopping processes.

The parallel resistance, which is an indicator for the active layer morphology (e.g., formation of “dead zones” from which no electrons can be extracted, and also to trap-assisted recombination [36]), tends to steadily increase with increased NC washing time, pointing towards an improved active layer morphology (*i.e.*, finer phase segregation and better charge extraction pathways). For post-synthetic NC treatment times exceeding the optimal duration, the parallel resistance tends to remain high in the resulting solar cells, but series resistance greatly increases, supposedly due to destruction of the NC surface by the washing procedure.

After performing a thermal annealing step on a hotplate inside a nitrogen filled glovebox for 10 min at 145 °C, a strong increase in *J_SC_* (and accordingly a decrease in *R_S_*) is observed especially for shorter washed QDs, which is probably mostly attributed to the enhanced intermolecular polymer-chain packing according to organic BHJ solar cell literature [36], but here a thermally induced *in situ* reduction of the NC ligand shell might also play a role. The series resistance for the longest washed NCs, however, strongly profits (despite still being of relatively high values) from thermal annealing, which might arise from a partial re-passivation of the NC surface. On the other side, the parallel resistance generally only appears to change after thermal annealing for solar cells containing shortly washed QDs, which we attribute to a higher morphological stability of the active layer of solar cells containing stronger ligand shell-size reduced QDs.

Briefly, after performing a first thermal annealing step on the solar cell, one can conclude whether one has to add to the NC washing time, or further reduce it for the next solar cell. This is, since the *J_SC_* increase is stronger after annealing for short-time-washed NCs, accompanied with a typical strong increase of *R_P_*. Moreover, solar cells containing short-time-washed NCs exhibit a higher V_OC_ after the same thermal annealing, compared to long-time-washed NCs containing cells.

### 2.5. Influence of NC Treatment on Solar Cell Long-Term Performance Stability

**Figure 9:**
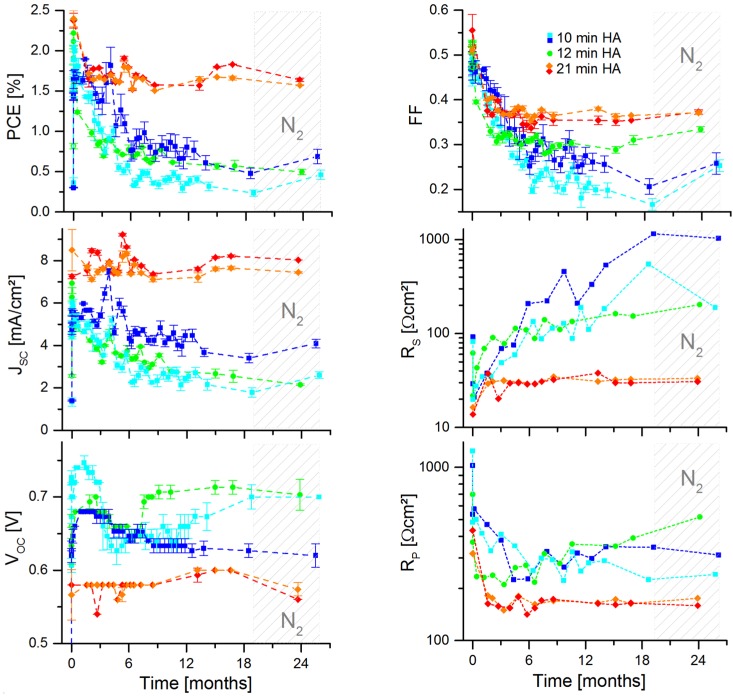
Parameters of PCPDTBT:CdSe QD solar cells from QDs washed by HA for 10 min (turquoise & blue squares), 12 min (green spherical points), and 21 min (orange & red rhombic points), stored in the dark inside a glovebox and periodically illuminated by a sun-simulator (AM 1.5 G spectrum) and characterized inside the same glovebox.

As we have previously shown, the post-synthetic NC treatment influences the immediate solar cell performance and the thermal stability of BHJ hybrid solar cells. As we will subsequently show, the post-synthetic NC treatment also influences the long-term performance of solar cells. Figure 9 shows the behavior of BHJ hybrid solar cells containing shorter (10 min and 12 min) and longer washed (21 min) CdSe QDs before being incorporated into the active layer.

As a result, we observe improved performance stability for solar cells containing longer-time-washed QDs, with a decrease of the PCE from 2.4% to 1.6% (−33%) compared to a decrease from 2.1% to 0.55% (−74%), during 2 years of investigation. The series resistance *R_S_* increases only slightly for long-time-washed NCs, but continuously increases for solar cells with shorter washed NCs. Possible explanations for this behavior would be the facilitated oxygen penetration through a supposedly less dense active layer for solar cells with higher ligand content, leading to increased oxidation of the CdSe QDs and of the polymer. However, the diffusion of ligands towards the electrodes, forming there an insulating layer would also be a possible explanation for the increasing *R_S_*. *R_P_* and *V_OC_* are already low for long-time-washed NCs, possibly due to more shunts within the active layer by presumably more aggregated NCs, and induced surface defects introduced by long NC washing, hence increasing charge recombination processes.

It is also worth mentioning that the short-circuit current has shown a clear dependency on the oxygen concentration, which fluctuated during the first 19 months around 25 ppm O_2_ with extremes of 0 ppm and 36 ppm, before the glovebox was continuously flushed with nitrogen for the following 5 months (the H_2_O concentration was of <5 ppm at all time). We have observed *J_SC_* to decrease with increasing oxygen concentration inside the glovebox, and *J_SC_* to be recovered after the oxygen concentration has decreased. This observed reversibility does not occur immediately, but rather slowly (within several days). Hence, the origin of *J_SC_* recovery might be due to oxygen depletion within the active layer by O_2_ diffusion.

### 2.6. Influence of NC Treatment on the Solar Cell Performance Stability in Air

We have also performed a short investigation of the influence the difference in NC treatment time has on the stability of non-encapsulated glass/ITO/PEDOT:PSS/PCPDTBT:CdSe/Al solar cells taken into air and measured under AM 1.5G illumination. Three different solar cells were used herein. The CdSe QDs utilized in this investigation received a HA-based treatment for 15 min (short washed) and 22 min (long washed). To this comparison, also an organic PC_61_BM:PCPDTBT solar cell, with the active layer composed out of a 2.5:1 weight ratio between PC_61_BM (Phenyl C_61_ butyric acid methyl ester, Sigma-Aldrich, Saint Louis, MS, USA, >99.5%) and PCPDTBT was added to specifically investigate the influence of the NCs.

**Figure 10:**
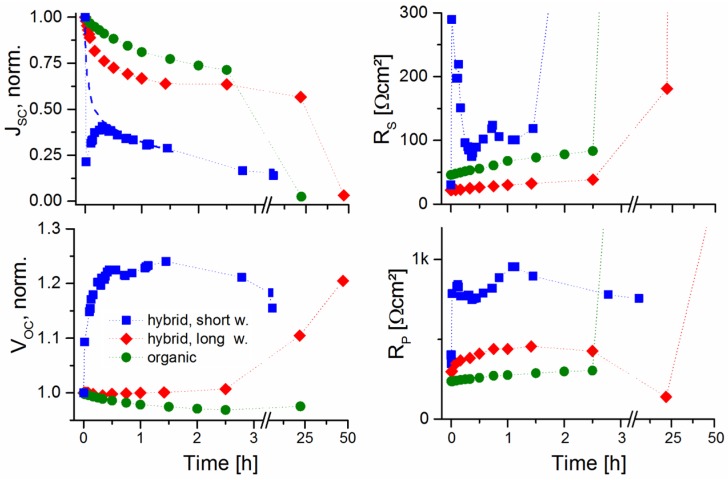
Development by time of short circuit current densities, series resistances, open circuit voltages, and parallel resistances for hybrid BHJ CdSe/PCPDTBT solar cells with short-time HA washed and long-time HA washed NCs, and for an organic BHJ PC_61_BM/PCPDTBT solar cell for comparison when taken into air. Since the J_SC_ of the solar cell containing short washed QDs displayed a fast initial decrease from its original value, an extrapolated graph has been added to guide the eye.

According to Figure 10, a decreasing *J_SC_* is observed for both hybrid and organic solar cells when taking the devices from the glovebox out into air. Possible reasons for the reduced *J_SC_* are the (partially) reversible uptake of oxygen within the polymer phase [37], the oxidation of the aluminum cathode [38], and the uptake of water into the hygroscopic Poly(3,4-ethylenedioxythiophene):poly(styrenesulfonic acid) (PEDOT:PSS) layer [39,40]. We also observe that the *J_SC_* decrease is more similar to the organic solar cell, when utilizing long-washed NCs; this might be due to the fact that the oxygen diffusion within the higher-density NC ligand depleted active layer is slowed down. The *V_OC_* shows a strong increase for hybrid solar cells containing short washed NCs, while a *V_OC_* increase is seen much later with long washed NCs, and not at all for organic solar cells—as has already been reported for organic solar cells [40,41]. Hence, we assume that the *V_OC_* increase in air is connected to the utilization of NCs, and possibly due to adsorption of H_2_O onto CdSe NCs.

## 3. Methods

### 3.1. Post-Synthetic NC Treatment

The post-synthetic NC treatment was performed in the following way: a portion of the synthesis product containing 1 mg of CdSe QDs was dissolved in 2.5 mL hexanoic acid (≥99.5%, Sigma-Aldrich, Saint Louis, MS, USA), stirred for several minutes in a snap-cap glass tube at 110 °C on a hot plate. Subsequently a double volume (compared to HA) of methanol (anhydrous, 99.8%, Sigma-Aldrich, Saint Louis, MS, USA) was added to reduce the QD concentration to 1/3rd of its initial value. The stirring was continued with the added methanol at 110 °C for half the stirring time compared to that in pure HA. Then, the dispersion was centrifuged by an Eppendorf MiniSpin^®^ plus centrifuge (Eppendorf AG, Hamburg, Germany) for 1 min at 14.5 krpm with the rotor-pre heated in a furnace to 90 °C in order to hinder the re-crystallization of the ligands. Afterwards the QDs were redispersed in chloroform (CHCl_3_) with a concentration of 2 mg/mL and stirred at 105 °C for 1 min. Consequently, triple the volume of methanol was added, and the NCs were further stirred for 3 min at 105 °C for precipitation. Afterwards, the NCs were collected by centrifugation for 30 s at 14.5 krpm. Chlorobenzene (anhydrous, 99.8%, Sigma-Aldrich, Saint-Louis, MS, USA) was then added to obtain a CdSe QD dispersion of 24 mg/mL. Hence, when mentioning the HA washing times throughout this publication, one must also consider the rest of the NC washing protocol subsequently executed, which additionally includes half the HA washing time with added MeOH, 1 min stirring in CHCl_3_, and finally 3 min stirring in MeOH. For example, 20 min of HA washing means: 20 min HA plus 10 min MeOH, centrifugation, redisperison, 1 min CHCl_3_ plus 3 min MeOH, centrifugation, and final redispersion in chlorobenzene (CB).

### 3.2. Solar Cell Fabrication

The obtained CdSe QD/CB dispersion was mixed in a weight ratio of 88:12 (QD:polymer) with a 20 mg/mL solution of PCPDTBT (Mn = 10–20 kDa, 1-Material, Dorval, Canada) in CB. The final ink was spun cast by a WS400-6NPP-Lite spin coater from Laurell Technologies (North Wales, PA, USA with 800 rpm for 30 s followed by a 60 s drying step at 1800 rpm, resulting in an active layer thickness of about 80 nm. The spin coating was done on a structured ≤10 Ω_sq_ ITO substrate from Präzisions Glas & Optik GmbH (Iserlohn, Germany), which was treated for 5 min with oxygen plasma and spin coated with Baytron AI4083 PEDOT:PSS from HC Starck GmbH (Goslar, Germany) at 2000 rpm for 30 s and dried for 20 min at 160 °C, to form a 70 nm thick hole blocking layer. After thermal evaporation of an 80 nm aluminum layer as electrode, the cells were thermally annealed for different times at 145 °C on a hot plate. The solar cells were usually annealed for 10 min and subsequently characterized. This procedure was repeated until the solar cell PCE started to decrease.

### 3.3. Solar Cell Characterization

The solar cells were characterized inside a nitrogen filled glovebox by a computer controlled Keithley 2602A source-meter (Keithley Instruments, Solon, OH, USA). The cells were individually illuminated by a LS0400 LOT-Oriel sun simulator (LOT-QuantumDesign GmbH, Darmstadt, Germany), housing a xenon lamp and using an AM 1.5G filter. The light intensity is adjusted by a calibrated silicon reference solar cell to match 100 mW/cm^2^.

### 3.4. Dynamic Light Scattering (DLS)

DLS measurements were performed with a Zetasizer Nanosizer ZS of Malvern Instruments Ltd (Malvern, UK). The investigated QDs were dispersed in chloroform to obtain a dispersion with an absorbance of 0.1 A.U. at the first excitonic peak. For the measurements a Hellma^®^ fluorescence cuvette out of Suprasil^®^ quartz glass (Hellma, Müllheim, Germany) was utilized.

## 4. Conclusions

The optimal NC treatment time before integration into BHJ hybrid solar cells can be determined by systematic analysis of the resulting solar cell device performances. NCs of higher PL QY require a longer post-synthetic NC treatment time. The optimal thermal annealing time for solar cell devices is different for different NC treatment times, and provides crucial information for tuning the NC treatment time in the right direction. Hybrid BHJ solar cells containing stronger ligand sphere reduced QDs exhibit an improved performance stability in the long term and in air. Further investigations would have to be conducted into the influence of the post-synthetic NC treatment on the active layer morphology and on the induced trap density on the NCs and inside the solar cell. We have shown in this publication that the QD synthesis procedure can lead to decisive influences on the optimal post-synthetic NC treatment before integration into hybrid BHJ solar cells and determine the overall solar cell performance. This might be one reason for the widely scattered results of groups working in this field and for QD batch to batch variations, since properties of nanomaterials are often determined by surface properties such as surface defects or ligand attachment and arrangement, which are hard to control and cannot be determined by TEM and UV-Vis spectroscopy, which are the methods of choice for QD characterization. We also believe that our findings for CdSe QDs are also of general importance for other QD systems and for the development of respective post-synthetic treatment protocols before integration into various applications.

## Figures and Tables

**Table 1:** Cd:Se precursor ratios, position of the photoluminescence (PL) brightpoint during the nanocrystal (NC) synthesis; average spectral PL peak position, PL full width at half maximum (FWHM), and average PL quantum yield (QY) of utilized quantum dots (QDs); and average power conversion efficiency (PCE) of hybrid bulk heterojunction poly[2,6-(4,4-bis-(2-ethylhexyl)-4H-cyclopenta[2,1-b;3,4-b’]dithiophene)-alt-4,7-(2,1,3-benzothiadiazole)]:CdSe (BHJ PCPDTBT:CdSe) QD solar cells found for the optimal post-synthetic hexanoic acid washing time of CdSe QDs.

Cd:Se Precursor Ratio	Brightpoint (min)	PL Peak Position (nm)	FWHM (nm)	PL QY (%)	Optimal HA Washing Time (min)	PCE with Optimal NC Treatment Time (%)
3:2	17	647 ± 7	30.7 ± 0.8	24 ± 5	21	2.4 ± 0.10
2:2	21	650 ± 3	27.0 ± 0.7	25 ± 5	18	2.8 ± 0.18
2:3	60	646 ± 5	32.2 ± 0.9	37 ± 8	60	1.7 ± 0.04

## Abbreviations

The following abbreviations are used in this manuscript:

NC	Nanocrystal
BHJ	Bulk heterojunction
PCPDTBT	(poly[2,6-(4,4-bis-(2-ethylhexyl)-4H-cyclopenta[2,1-b;3,4-b’]dithiophene)-alt-4,7-(2,1,3-benzothiadiazole)])
PEDOT:PSS	(Poly(3,4-ethylenedioxythiophene):poly(styrenesulfonic acid))
PCE	Power conversion efficiency
QD	Quantum dot
NR	Nanorod
MP	Multipod
HDA	Hexadecylamine
TOPO	Trioctylphosphine oxide
HA	Hexanoic acid
PL	Photoluminescence
Cd-SA	Cadmium-stearic acid
TOP-Se	Trioctylphosphine-selenid
UV-Vis	Ultraviolet-visible
FWHM	Full width at half maximum
CB	Chlorobenzene
MeOH	Methanol
DLS	Dynamic light scattering
A.U.	Absorbance units
ITO	Indium tin oxide
QY	Quantum yield
*V*_OC_	Open-circuit voltage
FF	Fill factor
*J*_SC_	Short-circuit current density
R_S_	Series resistance
R_P_	Parallel resistance

## References

[1] Zhou Y., Eck M., Krueger M. (2010). Bulk-heterojunction hybrid solar cells based on colloidal nanocrystals and conjugated polymers. Energy Environ. Sci..

[2] Greenham N.C., Peng X., Alivisatos A.P. (1996). Charge separation and transport in conjugated-polymer/semiconductor-nanocrystal composites studied by photoluminescence quenching and photoconductivity. Phys. Rev. B.

[3] Ren S., Chang L.-Y., Lim S.-K., Zhao J., Smith M., Zhao N., Bulović V., Bawendi M., Gradečak S. (2011). Inorganic–organic hybrid solar cell: Bridging quantum dots to conjugated polymer nanowires. Nano Lett..

[4] Greaney M.J., Araujo J., Burkhart B., Thompson B.C., Brutchey R.L. (2013). Novel semi-random and alternating copolymer hybrid solar cells utilizing CdSe multipods as versatile acceptors. Chem. Commun..

[5] Zhou R., Stalder R., Xie D., Cao W., Zheng Y., Yang Y., Plaisant M., Holloway P.H., Schanze K.S., Reynolds J.R. (2013). Enhancing the efficiency of solution-processed polymer: Colloidal nanocrystal hybrid photovoltaic cells using ethanedithiol treatment. ACS Nano.

[6] Eck M., van Pham C., Züfle S., Neukom M., Sessler M., Scheunemann D., Erdem E., Weber S., Borchert H., Ruhstaller B. (2014). Improved efficiency of bulk heterojunction hybrid solar cells by utilizing CdSe quantum dot-graphene nanocomposites. Phys. Chem. Chem. Phys..

[7] Han L., Qin D., Jiang X., Liu Y., Wang L., Chen J., Cao Y. (2006). Synthesis of high quality zinc-blende CdSe nanocrystals and their application in hybrid solar cells. Nanotechnology.

[8] Heinemann M.D., von Maydell K., Zutz F., Kolny-Olesiak J., Borchert H., Riedel I., Parisi J. (2009). Photo-induced charge transfer and relaxation of persistent charge carriers in polymer/nanocrystal composites for applications in hybrid solar cells. Adv. Funct. Mater..

[9] Olson J.D., Gray G.P., Carter S.A. (2009). Optimizing hybrid photovoltaics through annealing and ligand choice. Sol. Energy Mater. Sol. Cells.

[10] Dayal S., Reese M.O., Ferguson A.J., Ginley D.S., Rumbles G., Kopidakis N. (2010). The effect of nanoparticle shape on the photocarrier dynamics and photovoltaic device performance of poly(3-hexylthiophene):CdSe nanoparticle bulk heterojunction solar cells. Adv. Funct. Mater..

[11] Zhou Y., Riehle F.S., Yuan Y., Schleiermacher H.-F., Niggemann M., Urban G.A., Krueger M. (2010). Improved efficiency of hybrid solar cells based on non-ligand-exchanged CdSe quantum dots and poly(3-hexylthiophene). Appl. Phys. Lett..

[12] Zhou Y., Eck M., Veit C., Zimmermann B., Rauscher F., Niyamakom P., Yilmaz S., Dumsch I., Allard S., Scherf U. (2011). Efficiency enhancement for bulk-heterojunction hybrid solar cells based on acid treated CdSe quantum dots and low bandgap polymer PCPDTBT. Sol. Energy Mater. Sol. Cells.

[13] Huynh W.U., Dittmer J.J., Alivisatos A.P. (2002). Hybrid nanorod-polymer solar cells. Science.

[14] Sun B., Greenham N.C. (2006). Improved efficiency of photovoltaics based on CdSe nanorods and poly(3-hexylthiophene) nanofibers. Phys. Chem. Chem. Phys..

[15] Celik D., Krueger M., Veit C., Schleiermacher H.F., Zimmermann B., Allard S., Dumsch I., Scherf U., Rauscher F., Niyamakom P. (2012). Performance enhancement of CdSe nanorod-polymer based hybrid solar cells utilizing a novel combination of post-synthetic nanoparticle surface treatments. Sol. Energy Mater. Sol. Cells.

[16] Sun B.Q., Marx E., Greenham N.C. (2003). Photovoltaic devices using blends of branched CdSe nanoparticles and conjugated polymers. Nano Lett..

[17] Sun B., Snaith H.J., Dhoot A.S., Westenhoff S., Greenham N.C. (2005). Vertically segregated hybrid blends for photovoltaic devices with improved efficiency. J. Appl. Phys..

[18] Zhou Y., Li Y., Zhong H., Hou J., Ding Y., Yang C., Li Y. (2006). Hybrid nanocrystal/polymer solar cells based on tetrapod-shaped CdSe*_x_* Te_1−*x*_ nanocrystals. Nanotechnology.

[19] Wang P., Abrusci A., Wong H.M.P., Svensson M., Andersson M.R., Greenham N.C. (2006). Photoinduced charge transfer and efficient solar energy conversion in a blend of a red polyfluorene copolymer with CdSe nanoparticles. Nano Lett..

[20] Dayal S., Kopidakis N., Olson D.C., Ginley D.S., Rumbles G. (2010). Photovoltaic devices with a low band gap polymer and CdSe nanostructures exceeding 3% efficiency. Nano Lett..

[21] Dennler G., Scharber M.C., Brabec C.J. (2009). Polymer-fullerene bulk-heterojunction solar cells. Adv. Mater..

[22] Peet J., Kim J.Y., Coates N.E., Ma W.L., Moses D., Heeger A.J., Bazan G.C. (2007). Efficiency enhancement in low-bandgap polymer solar cells by processing with alkane dithiols. Nat. Mater..

[23] Zillner E., Fengler S., Niyamakom P., Rauscher F., Köhler K., Dittrich T. (2012). Role of ligand exchange at CdSe quantum dot layers for charge separation. J. Phys. Chem. C.

[24] Riehle F.S. (2013). The Rational Synthesis Of Defect-Free CdE (E=S,Se) Nanocrystals. From Precursor Reactivity to Surface Stability. Ph.D. Thesis.

[25] Gao F., Li Z., Wang J., Rao A., Howard I.A., Abrusci A., Massip S., McNeill C.R., Greenham N.C. (2014). Trap-induced losses in hybrid photovoltaics. ACS Nano.

[26] Zhu L., Richardson B.J., Yu Q. (2016). Inverted hybrid CdSe-polymer solar cells adopting PEDOT:PSS/MoO_3_ as dual hole transport layers. Phys. Chem. Chem. Phys..

[27] Ramar M., Suman C.K., Manimozhi R., Ahamad R., Srivastava R. (2014). Study of Schottky contact in binary and ternary hybrid CdSe quantum dot solar cells. RSC Adv..

[28] Lek J.Y., Lam Y.M., Niziol J., Marzec M. (2012). Understanding polycarbazole-based polymer: CdSe hybrid solar cells. Nanotechnology.

[29] Kippelen B., Brédas J.-L. (2009). Organic photovoltaics. Energy Environ. Sci..

[30] Steinmann V., Kronenberg N.M., Lenze M.R., Graf S.M., Hertel D., Meerholz K., Bürckstümmer H., Tulyakova E.V., Würthner F. (2011). Simple, highly efficient vacuum-processed bulk heterojunction solar cells based on merocyanine dyes. Adv. Energy Mater..

[31] Yuan Y., Riehle F.-S., Gu H., Thomann R., Urban G., Krueger M. (2010). Critical parameters for the scale-up synthesis of quantum dots. J. Nanosci. Nanotechnol..

[32] Qu L., Peng X. (2002). Control of photoluminescence properties of CdSe nanocrystals in growth. J. Am. Chem. Soc..

[33] Krueger M., Eck M., Zhou Y., Riehle F.-S. (2012). Semiconducting nanocrystal/conjugated polymer composites for applications in hybrid polymer solar cells. Semiconducting Polymer Composites.

[34] Eichhöfer A., Hänisch C.V., Jacobsohn M., Banin U., Karim A., Merhari L., Norris D.J., Rogers J.A., Xia Y. (2000). Dynamic light scattering at CdSe nanocrystals and CdSe cluster-molecules. Proceedings of the MRS Fall Meeting 2000.

[35] Shen Y., Li K., Majumdar N., Campbell J.C., Gupta M.C. (2011). Bulk and contact resistance in P3HT:PCBM heterojunction solar cells. Sol. Energy Mater. Sol. Cells.

[36] Kim M.-S., Kim B.-G., Kim J. (2009). Effective variables to control the fill factor of organic photovoltaic cells. ACS Appl. Mater. Interfaces.

[37] Schafferhans J., Baumann A., Wagenpfahl A., Deibel C., Dyakonov V. (2010). Oxygen doping of P3HT:PCBM blends: Influence on trap states, charge carrier mobility and solar cell performance. Org. Electron..

[38] Tang J., Wang X., Brzozowski L., Barkhouse D.A., Debnath R., Levina L., Sargent E.H. (2010). Schottky quantum dot solar cells stable in air under solar illumination. Adv. Mater..

[39] Kwon S., Lim K.-G., Shim M., Moon H.C., Park J., Jeon G., Shin J., Cho K., Lee T.-W., Kim J.K. (2013). Air-stable inverted structure of hybrid solar cells using a cesium-doped ZnO electron transport layer prepared by a sol–gel process. J. Mater. Chem. A.

[40] Züfle S., Neukom M.T., Altazin S., Zinggeler M., Chrapa M., Offermans T., Ruhstaller B. (2015). An effective area approach to model lateral degradation in organic solar cells. Adv. Energy Mater..

[41] Kawano K., Pacios R., Poplavskyy D., Nelson J., Bradley D.D., Durrant J.R. (2006). Degradation of organic solar cells due to air exposure. Sol. Energy Mater. Sol. Cells.

